# Regulation of Proteome Maintenance Gene Expression by Activators of Peroxisome Proliferator-Activated Receptor *α*

**DOI:** 10.1155/2010/727194

**Published:** 2011-01-17

**Authors:** Hongzu Ren, Beena Vallanat, Holly M. Brown-Borg, Richard Currie, J. Christopher Corton

**Affiliations:** ^1^NHEERL Toxicogenomics Core, US EPA, Research Triangle Park, NC 27711, USA; ^2^Department of Pharmacology, Physiology and Therapeutics, University of North Dakota, School of Medicine, 501 N. Columbia Road, Grand Forks, ND 58203-2817, USA; ^3^Syngenta Central Toxicology Laboratory, Alderley Park, Cheshire SK104TJ, UK; ^4^Integrated Systems Toxicology Division, National Health and Environmental Effects Research Lab, US Environmental Protection Agency, Research Triangle Park, NC 27711, USA

## Abstract

The nuclear receptor peroxisome proliferator-activated receptor *α* (PPAR*α*) is activated by a large number of xenobiotic and hypolipidemic compounds called peroxisome proliferator chemicals (PPCs). One agonist of PPAR*α* (WY-14,643) regulates responses in the mouse liver to chemical stress in part by altering expression of genes involved in proteome maintenance (PM) including protein chaperones in the heat shock protein (*Hsp*) family and proteasomal genes (*Psm*) involved in proteolysis. We hypothesized that other PPAR*α* activators including diverse hypolipidemic and xenobiotic compounds also regulate PM genes in the rat and mouse liver. We examined the expression of PM genes in rat and mouse liver after exposure to 7 different PPCs (WY-14,643, clofibrate, fenofibrate, valproic acid, di-(2-ethylhexyl) phthalate, perfluorooctanoic acid, and perfluorooctane sulfonate) using Affymetrix microarrays. In rats and mice, 174 or 380 PM genes, respectively, were regulated by at least one PPC. The transcriptional changes were, for the most part, dependent on PPAR*α*, as most changes were not observed in similarly treated PPAR*α*-null mice and the changes were not consistently observed in rats treated with activators of the nuclear receptors CAR or PXR. In rats and mice, PM gene expression exhibited differences compared to typical direct targets of PPAR*α* (e.g., *Cyp4a* family members). PM gene expression was usually delayed and in some cases, it was transient. Dose-response characterization of protein expression showed that Hsp86 and Hsp110 proteins were induced only at higher doses. These studies demonstrate that PPAR*α*, activated by diverse PPC, regulates the expression of a large number of genes involved in protein folding and degradation and support an expanded role for PPAR*α* in the regulation of genes that protect the proteome.

## 1. Introduction

Peroxisome proliferator chemicals (PPCs) are a large class of structurally heterogeneous pharmaceutical and industrial chemicals originally identified as inducers of the size and number of peroxisomes in rodent livers. The peroxisome proliferator-activated receptor family is a subset of the nuclear receptor superfamily and includes three family members (PPAR*α*, *β*, and *γ*). The PPAR*α* subtype plays a dominant role in mediating the effects of hypolipidemic and xenobiotic PPC in the liver [1]. Activation of PPAR*α* results in a predictable set of responses in the livers of rats and mice, including hepatocyte peroxisome proliferation, hepatomegaly, hepatocyte hyperplasia, and increased incidence of liver tumors [2]. These responses require a functional PPAR*α*, since PPAR*α*-null mice exposed to the PPAR*α* agonists WY or bezafibrate lack all of these responses [3–5]. PPAR*α* controls these phenotypic responses by regulating a large number of genes in the liver including those involved in lipid homeostasis such as fatty acid oxidation and peroxisome assembly. 

Various physical or chemical stressors can produce disease states in which proteins are damaged or misfolded in part through increases in oxidative stress. Many endogenous pathways are activated to restore cellular homeostasis, including stabilization of unfolded proteins to prevent aggregation as well as removal of damaged or excess proteins through proteolysis. Stabilization of unfolded proteins is performed by molecular chaperones that assist in the folding of nascent polypeptides. Many chaperone genes exhibit increased expression after exposure to a wide variety of stimuli including chemical exposure or increased temperatures and are thus called heat shock (HS) protein (*Hsp*) genes [6–8]. These proteins play key roles in a number of human diseases [9] and are essential for cellular survival under physical or chemical exposure conditions that increase oxidative stress [10, 11]. Additional guardians of the proteome include the genes encoding components of the proteasome. The proteasome carries out ubiquitin-dependent and -independent proteolysis of damaged proteins [12]. The 26S proteasome consists of a 20S core and two 19S regulatory particles containing a total of 28 subunits. Proteasomal (*Psm*) gene expression can be induced by treating cells with proteasomal inhibitors [13].

There is compelling evidence that PPAR*α* protects multiple tissues from oxidative stress induced by chemical insults [14]. The hypolipidemic drug and PPC, clofibrate, protects the liver from damage from the cytotoxicant acetaminophen in wild-type but not PPAR*α*-null mice [15]. Compared to wild-type mice, untreated PPAR*α*-null mice or primary hepatocytes isolated from PPAR*α*-null mice were more sensitive to carbon tetrachloride-, paraquat- or cadmium-induced toxicity [16]. The beneficial effects of caloric restriction in protecting the liver from cytotoxicant-induced liver injury were shown to depend on PPAR*α* [17]. In the kidney, PPAR*α*-null mice were more sensitive to damage after ischemia-reperfusion injury [18], and prior exposure of wild-type mice to PPC reduces the injury [19]. Our previous microarray studies identified PM genes regulated by the PPAR*α* agonist WY-14,643 (WY) including those involved in protein folding (e.g., *Hsp* genes) as well as ubiquitin-dependent and -independent proteolytic processing through the proteasome (e.g., *Psm* genes) [16]. Altered regulation of these genes by PPC could help to explain why PPC exposure through PPAR*α* helps to protect tissues from environmental stressors.

In the present study, we hypothesized that PPAR*α* activators other than WY also regulate PM genes in the rat and mouse liver. We examined the expression of PM genes in rat and mouse liver after exposure to 7 different PPC (WY, clofibrate (CLO), fenofibrate (FENO), valproic acid (VPA), di-(2-ethylhexyl)phthalate (DEHP), perfluorooctanoic acid (PFOA) and perfluorooctane sulfonate (PFOS)) using Affymetrix microarrays from published studies. We show that both therapeutic and environmentally relevant PPC exposure has a dramatic impact on PM gene expression in the rat and mouse liver. Although most of the changes were PPAR*α*-dependent, there were differences in their temporal and dose-dependent regulation compared to typical PPAR*α* target genes involved in fatty acid oxidation. Our findings suggest PPAR*α* is a major regulator of PM genes that have an impact on stress responses in the liver.

## 2. Materials and Methods

### 2.1. Animal Studies for Chaperone Protein Expression

The first study was carried out at CIIT, Centers for Health Research, Research Triangle Park, NC utilized wild-type and PPAR*α*-null male mice 9–12 weeks of age on a mixed SV129/C57BL/6J background and have been described previously [20]. Control and treated mice were provided with NIH-07 rodent chow (Zeigler Brothers, Gardeners, PA) and deionized, filtered water *ad libitum*. Lighting was on a 12-hr light/dark cycle. Wild-type and PPAR*α*-null mice were given seven daily gavage doses of 0.1% methyl cellulose control (Sigma Chemical Co., St. Louis, MO), or di-(2-ethylhexyl)phthalate (DEHP) (1000 mg/kg/bw/day) and sacrificed 24 hrs after the last dose. 

The second study (NTP study number TOX-60) was carried out at Battelle (Columbus, OH) under a contract from the National Toxicology Program. Male B6C3F1 mice at 4–6 weeks of age were obtained from Taconic Farms, Inc. (Germantown, NY). The feed was NTP-2000 in meal form (Zeigler Brothers, Inc., Gardners, PA) and the drinking water was from the City of Columbus municipal supply. Both feed and water were supplied ad libitum. At 7-8 weeks of age, the mice received in their feed WY at 0, 5, 10, 50, 100, or 500 ppm. The mice were euthanized after 6 days of exposure to WY. Portions of the livers were rapidly snap-frozen in liquid nitrogen and stored at −70°C until analysis. All animal studies were conducted under federal guidelines for the use and care of laboratory animals and were approved by Institutional Animal Care and Use Committees.

### 2.2. Animal Studies Used for Microarray Analysis

The experiments related to clofibrate- (CLO-) and valproic acid- (VPA-) treated rats were described in Jolly et al. [21]. Briefly, male Sprague-Dawley rats (*n* = 5) were given a single dose of CLO or VPA at the level of 1,000 mg/kg and 2,000 mg/kg, respectively. Animals were killed at 4, 24, and 48 hrs after exposure. Eleven-to-12-week-old male Sprague-Dawley rats were dosed with 20 or 10 mg/kg/day PFOA or PFOS, respectively, in an aqueous solution of 15% Alkamuls EL-620 for 2 days and sacrificed 24 hours later as described in Martin et al. [22]. The animal study of fenofibrate (FENO) was described in Sanderson et al. [23]. Male pure-bred SV129 and PPAR*α*-null mice (2–6 months of age) on a SV129 background were used in the experiments. Fenofibrate was given by gavage (10 mg/ml suspension in 0.5% carboxymethyl cellulose). Animals were sacrificed 6 hours after treatment. Four wild-type and PPAR*α*-null male mice (129S1/SvlmJ wild-type and PPAR*α*-null) per group (6 months of age) were dosed by gavage for 7 consecutive days with PFOA (3 mg/kg/day) in distilled water as described in Rosen et al. [24]. At the end of the dosing period, animals were euthanized by CO_2_ asphyxiation and liver tissue was collected for preparation of total RNA.

### 2.3. Western Blot Analysis

Liver lysates were prepared in 250 mM sucrose, 10 mM Tris-HCl, pH 7.4, and 1 mM EDTA with protease inhibitors (0.2 mM phenylmethylsulfonyl fluoride, 0.1% aprotinin, 1 *μ*g/ml pepstatin, 1 *μ*g/ml leupeptin) as previously described [25]. Fifty *μ*g hepatocyte whole cell lysate was subjected to 12% sodium dodecyl sulfate-polyacrylamide gel electrophoresis followed by transfer to nitrocellulose membranes. Immunoblots were developed using primary antibodies against acyl-CoA oxidase (ACO) (a kind gift from Stefan Alexson, Huddinge University Hospital, Huddinge, Sweden), HS proteins (Santa Cruz Biotechnology, Santa Cruz, CA; StressGen, Victoria, B.C., Canada) or CYP4A (GenTest, Waltham, MA) and appropriate secondary antibodies conjugated with horseradish peroxidase (Santa Cruz Biotechnology) in the presence of chemiluminescent substrate ECL (Amersham, Piscataway, NJ). Blots were quantitated using Gel-Pro (MediaCybernetics, Silver Spring, MD). Most antibodies recognized only one major band with the expected size. Antibodies to TCP1*η* routinely gave 2 bands: a ~60 kDa representing the full-length protein and a possible fragment of ~40 kDa. In this study we report the levels of the full-length TCP*η* and ACO protein (ACO-A). The expression of ACO-B protein, the 52 kDa processed form of ACO-A [26] was also measured. There were 3 animals per treatment group. Variability is expressed as standard error of the mean. Means and S.E. (*n* = 3) for western data were calculated by Student's *t-*test. The level of significance was set at *P* ≤ .05.

### 2.4. Analysis of Microarray Data

A summary of the microarray studies is shown in Table 1. The doses selected in these studies would be expected to elicit close to a maximal transcriptional response. The raw data files analyzed in this project (.cel files from Affymetrix DNA chips) were either downloaded from Gene Expression Omnibus (GEO) or communicated through the original authors. All of the Affymetrix (Santa Clara, CA)  .cel files were first analyzed by Bioconductor SimpleAffy to assess data quality [27]. All .cel files passed this QC step. Data (.cel files) were analyzed and statistically filtered using Rosetta Resolver version 7.1 software (Rosetta Inpharmatics, Kirkland, WA). The background correction was done by Resolver's specific data processing pipeline called Affymetrix Rosetta-Intensity Profile Builder. Statistically significant genes were identified using one-way ANOVA with a false discovery rate (Benjamini-Hochberg test) of ≤0.05 followed by a post hoc test (Scheffe) for significance. Fold-change values <1.5 were removed. As most of the experiments in rats used the RG-U34A array, we compared profiles in the RG-U34A annotation file from Affymetrix (http://www.affymetrix.com/analysis/index.affx). We identified probeset IDs (a total of 8799) on the U34A chip that exhibited sequence similarity with those on the RAE230_2 chip using the “good match” comparison and then built fold-change values for those genes from the RAE230_2 chip which were altered significantly. Heat maps were generated using Eisen Lab Cluster and Treeview software (http://rana.lbl.gov/EisenSoftware.htm). A detailed description of each experiment is available through Gene Expression Omnibus at the National Center for Biotechnology Information at http://www.ncbi.nlm.nih.gov/geo/, as accession numbers indicated in Table 1. 

PM genes were identified using the following gene ontology identifiers: 0031072:heat shock protein binding, 0006457:protein folding, 009408:response to heat, 0051085:chaperone cofactor-dependent protein folding, 0006950:response to stress, 0006983:endoplasmic reticulum overload, 0006512:ubiquitin cycle, and 0006511:ubiquitin-dependent protein catabolic process. A number of proteasome genes (*Psm* family) not linked to GO identifiers were also included. 

## 3. Results

Our previous experiments indicated that a PPAR*α* agonist (WY) alters the expression of PM genes in the mouse liver including those involved in protein folding and protein degradation [16]. To determine if other PPAR*α* agonists have similar activities, we examined transcript profiles in rat and mouse liver after treatment with compounds that possess PPAR*α* agonist-like activities including three hypolipidemic compounds (WY, clofibrate (CLO), fenofibrate (FENO)), an antiepilepsy drug (valproic acid, VPA), and three environmentally relevant chemicals (the plasticizer, di-(2-ethylhexyl)phthalate (DEHP) and the surfactant processing aids, perfluorooctanoic acid (PFOA) and perfluorooctane sulfonate (PFOS) (Table 1). PM genes were identified using gene ontology (GO) identifiers (e.g., protein folding; response to stress including endoplasmic reticulum overload; ubiquitin-dependent protein catabolic process). 

### 3.1. Altered Expression of Proteome Maintenance Genes in the Rat and Mouse Liver after Exposure to Diverse PPC

**Figure 1:**
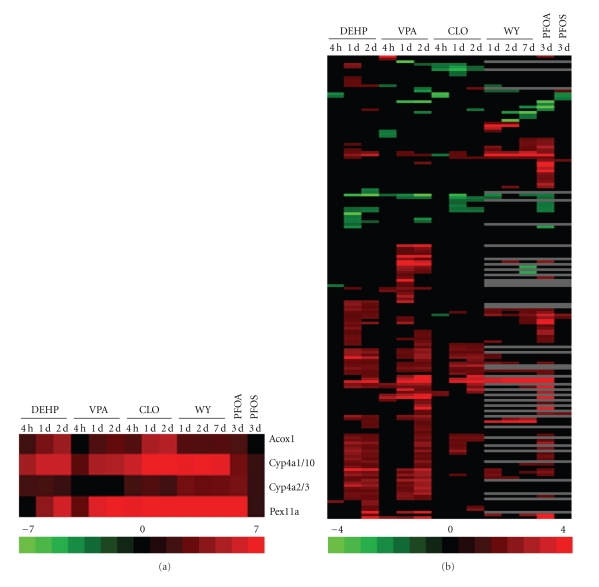
Altered expression of proteome maintenance genes by diverse PPC in rat liver. Male rats were treated with DEHP, VPA, CLO, WY, PFOA, or PFOS for the indicated times. Hepatic mRNA transcripts were assessed using Affymetrix arrays. Genes involved in PM including protein chaperones were identified as described in Section 2. (a) Positive control genes. (b) Expression of all PM genes altered by at least one of the treatments. Genes were clustered using one-dimensional hierarchical clustering. All genes are found in the Supplementary Material. Red: up-regulation; green: down-regulation; black: no change; grey: no data. The intensity scales indicates fold-change due to chemical exposure relative to controls. Abbreviations: WY: WY-14,643; DEHP: di-(2-ethylhexyl)phthalate; PFOA: perfluorooctanoic acid; VPA: valproic acid; CLO: clofibrate; PFOS: perfluorooctane sulfonate.

**Figure 2:**
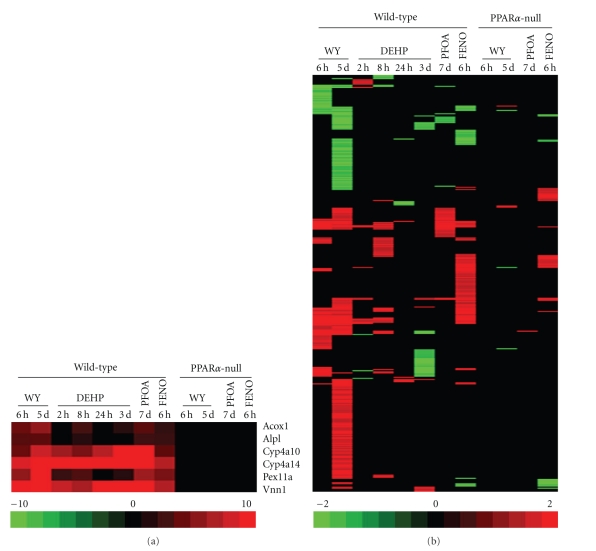
Altered expression of proteome maintenance genes by diverse PPC is predominantly PPAR*α* dependent. Wild-type or PPAR*α*-null mice were treated with WY, DEHP, PFOA, or FENO for the indicated times. Hepatic mRNA transcripts were assessed using full-genome Affymetrix arrays. Genes involved in PM including protein chaperones were identified as described in Section 2. (a) Positive control genes. (b) Expression of all PM genes altered by at least one of the treatments. Genes were clustered using one-dimensional hierarchical clustering. All genes are found in Supplemental Material. Red: up-regulation; green: down-regulation; black: no change. The intensity scales indicate fold-change due to chemical exposure relative to controls. Abbreviations: WY: WY-14,643; DEHP: di-(2-ethylhexyl)phthalate; PFOA: perfluorooctanoic acid; FENO: fenofibrate.

We examined gene expression in the livers of rats and mice treated with PPC. In both species, PPC increased expression of genes known to be regulated by PPAR*α* including those involved in fatty acid oxidation such as *Cyp4a* family members, acyl-CoA oxidase 1 (*Acox1*) and peroxisome assembly genes, for example, *Pex11a *(Figures 1(a) and 2(a)). The global expression of all PM genes in the rat and mouse liver is shown in Figures 1(b) and 2(b), respectively. Out of a total of 288 PM probe sets identified in the rat, 174 were altered by one or more of the 14 treatment conditions (Supplemental File 1). Likewise, out of the total of 1597 PM probe sets examined in the mouse, 382 were altered by one or more of the 12 treatment conditions (see Supplementary Material available online at doi:10.1155/2010/727194). 

There were a number of similarities exhibited by the PM genes in both species. First, the altered genes were dominated by those that were upregulated after exposure. The upregulated genes outnumbered the downregulated genes ~2 to 1. Second, the PM genes exhibited a delay in altered expression compared to the known direct targets of PPAR*α*. The direct targets of PPAR*α* were increased as soon as 4 hrs (rat) or 6 hr (mouse) after initial exposure. In contrast, almost none of the PM genes in rats were altered at 4 hrs and some required up to 2 days of exposure before expression changes were observed. Most of the expression changes that occurred after WY exposure in the mouse liver were observed at 5 days but not 6 hrs. Third, a number of PM genes exhibited transient changes compared to the constant expression of the direct targets of PPAR*α*. Genes were induced by DEHP, VPA or CLO at 1 but not 2 days; in the mouse liver, a subset of genes were induced by DEHP at 8 hrs but not at any other time. Fourth, there were PPC-specific effects. In the rat VPA, WY and PFOA all increased unique sets of genes. Another set of genes was increased by DEHP at day 1 but decreased by VPA and CLO. In the mouse liver WY, DEHP and FENO altered unique subsets of genes. 

The transcriptional changes were, for the most part dependent on PPAR*α*, as most changes were not observed in similarly treated PPAR*α*-null mice. The exceptions included the altered regulation of 6 genes by WY for 5 days, 1 gene by PFOA for 7 days and 41 genes by fenofibrate for 6 hrs. Although fenofibrate is considered a PPAR*α* agonist, there is evidence that other fibrates can activate PPAR*γ* in transactivation assays [1], and pan-PPAR activation may contribute to PPAR*α*-independent regulation of a subset of the PM genes. Overall, these results indicate that both fatty acid metabolizing and PM genes were dependent on PPAR*α* for altered regulation by PPC. The PM genes exhibited unique characteristics in their pattern of expression.

### 3.2. Regulation of Proteasomal Genes by PPC

**Figure 3:**
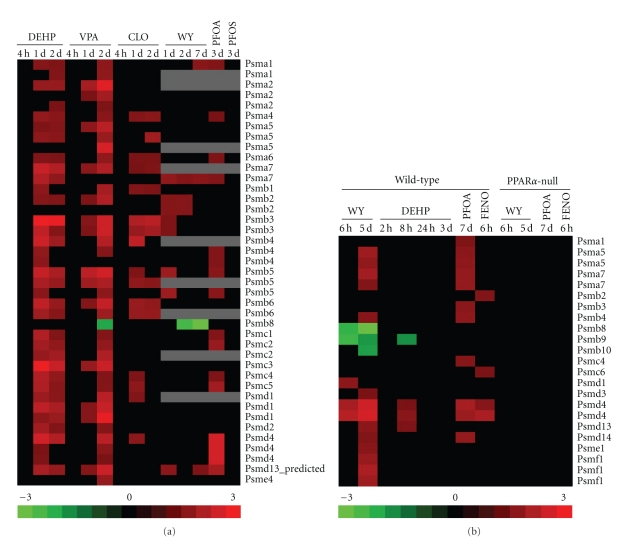
Expression of the proteasomal genes in the rat and mouse liver after PPC treatment. Proteasomal gene expression was examined in the (a) rat liver or (b) mouse liver after PPC exposure using the studies described in Figures 1 and 2. Genes which exhibited altered expression in at least one of the treatments are shown. Genes are presented in alphabetical order. Many genes were represented by more than one probe set.

A large number of genes encoding components of the proteasome were altered by WY [16]. We examined the expression of the proteasomal genes (*Psm*) as well as those known to be involved in ubiquitin-dependent proteolysis after PPC exposure (Figures 3(a) and 3(b)). In both species all of the *Psm* genes which exhibited altered regulation were upregulated except those that are components of the immunoproteasome (i.e., *Psmb8* in rats and *Psmb8, Psmb9 *and *Psmb10* in mice). DEHP and VPA in rats and WY and PFOA in mice altered the largest number of *Psm *genes. In contrast, DEHP in mice transiently altered only a small set of *Psm* genes. The changes were never observed earlier than 1 day in rats. DEHP, VPA and PFOA in rats and WY and PFOA in mice altered subunits of both the catalytic core (20S proteasome) and the ATP-dependent regulatory core (19S proteasome), whereas CLO and WY in rats preferentially altered 20S components. There were a number of genes that were altered in both species including *Psma1, Psma5, Psma7, Psmb2, Psmb3, Psmb4, Psmb8, Psmc4, Psmd1, Psmd4, *and* Psmd13*. All of the *Psm* genes altered by PPC in mice were PPAR*α* dependent. 

In an examination of the ubiquitination machinery, PPC altered 8 probesets (6 genes) in rat liver and 48 probesets (35 genes) in mouse liver (data not shown; see Supplemenary Material). None of the rat genes were altered by more than two PPC and in the mouse liver, only one gene (*Usp38*) was altered by three out of the four PPCs. All but six of the ubiquitination genes were PPAR*α* dependent. These findings extend the results from our earlier study and show that diverse PPCs coordinately alter the expression of the *Psm* genes in a PPAR*α*-dependent manner.

### 3.3. Regulation of Protein Chaperone Genes by PPC

**Figure 4:**
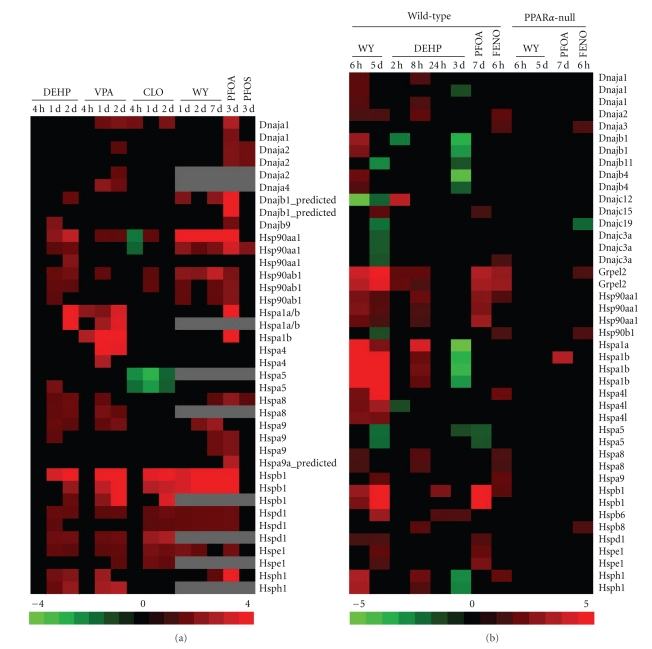
Expression of the protein chaperone genes in the rat and mouse liver after PPC treatment. Protein chaperone gene expression was examined in the (a) rat liver or (b) mouse liver after PPC exposure using the studies described in Figures 1 and 2. Genes which exhibited altered expression in at least one of the treatments are shown. Genes are presented in alphabetical order. Many genes were represented by more than one probe set.

We examined the expression of protein chaperone genes after PPC exposure (Figures 4(a) and 4(b)). Almost all of the chaperone genes were upregulated by PPC in rat liver and by WY in the mouse liver. There were many genes that were regulated by more than half of the PPC in rat liver including *Hsp90aa1, Hsp90ab1, Hspa8, Hspb1, Hspd1, Hspe1, Hspa9, *and* Hsph1*. In the mouse liver most of the PPC regulated a smaller set of genes including *Dnaja2, Grpel2, Hsp90aa1, Hspa4l, Hspa8, Hspb1,* and *Hsph1*. Many genes were commonly regulated in both species by at least half of the PPC (*Dnaja1, Dnaja2, Hsp90aa1, Hspa1a/b, Hspa8, Hspb1, Hspd1, Hspe1, Hspa9,* and * Hsph1*). Some of the changes were transient, exhibiting attenuated or no regulation after long-term exposure. For WY (Figure 4(b)), these included *Dnaja1, Dnajb1, Dnajb4, Hspa1a, Hspa1b, Hspa8, Hsph1*, and *Hsp90aa1 *and for DEHP, these included* Dnaja1, Dnaja2, Dnajc12, Grpel2, Hsp90aa1, Hspb1, *and *Hspb8*. There were a number of chaperone genes that were uniquely regulated by VPA (*Dnaja4, Hspa4*), PFOA (*Hspa9a-predicted*) or both (*Hspa1b*) in rat liver. Exposure to DEHP in the mouse for 3 days gave a unique pattern of changes in which *Dnaja1, Dnajb1, Dnajb4, Hspa1a, Hspa1b, *and* Hsph1 *were downregulated at 3 days compared to the consistent up-regulation by the other PPC. Furthermore, three of these genes (*Hspa1a, Hspa1b, Hsph1*) were upregulated after shorter-term exposure. These results indicate that multiple and possibly competing mechanisms may be regulating these genes after DEHP exposure, different from that of other PPC. In the mouse liver, most of the genes required PPAR*α* for altered expression except for *Dnaja3, Grpel2, *and *Hsp90b1* that appeared to be regulated similarly by FENO in wild-type and PPAR*α*-null mice.

### 3.4. Expression of Chaperone Proteins in Mouse Liver after PPC Exposure

**Figure 5:**
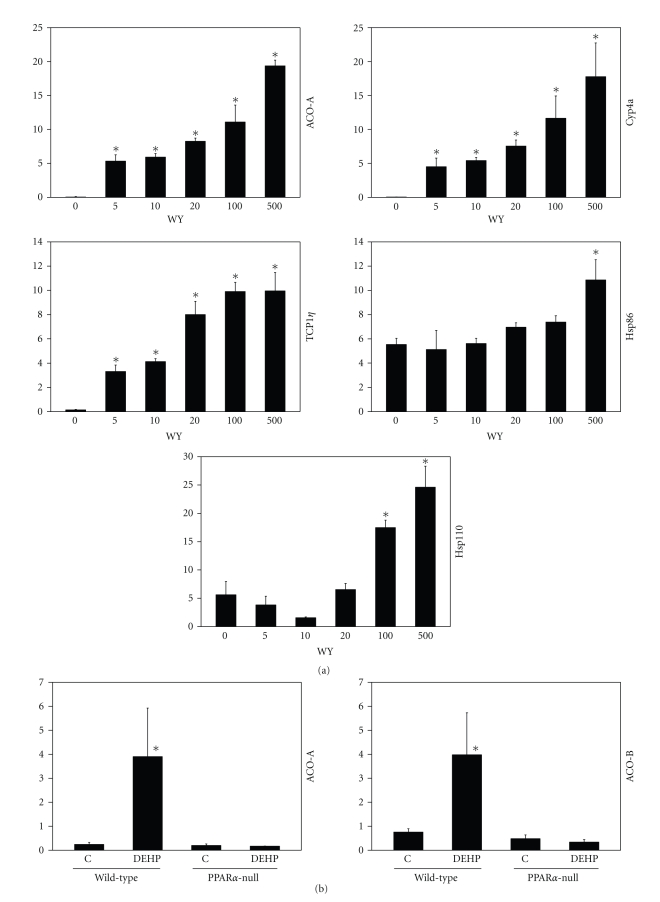

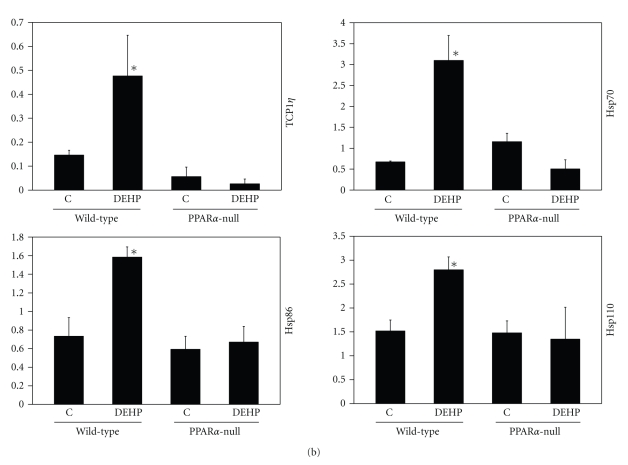
Expression of chaperone proteins after PPC treatment. (a) Protein expression in the livers of mice treated with different doses of WY. B6C3F1 mice were treated with the indicated concentrations of WY in the diet for 6 days. Protein expression was assessed by Westerns using primary antibodies against the indicated proteins. Blots were quantitated as described in Section 2. (b) Protein expression in livers of wild-type and PPAR*α*-null mice after exposure to DEHP. Proteins were extracted from the livers of wild-type or PPAR*α*-null mice given 7 consecutive doses of DEHP (1000 mg/kg day). There were 3 animals per treatment group. Variability is expressed as standard error of the mean. Means and S.E. (*n* = 3) for western data were calculated by Student's *t*-test. The level of significance was set at *P* ≤ .05 and significance is indicated with ∗.

We examined expression of protein chaperones in protein extracts from livers of mice given five different doses of WY for 6 days or DEHP at one dose level for 7 days. Given the transcriptional increases in chaperonin-containing T-complex 1 (Tcp-1) family members *Cct3, Cct4, Cct7,* and *Cct8 *after exposure to PPC in wild-type mice (Supplemental Material), we also examined the expression of Tcp1*η* protein. Dose-dependent increases in Tcp1*η*, Hsp86, Cyp4A, ACO-A and Hsp110 were observed in WY-treated wild-type B6C3F1 mice (Figure 5(a)). The chaperones exhibited dissimilar dose-dependent inductions as TCP1*η* was induced at lower doses similar to the direct PPAR*α* targets ACO-A and Cyp4a, whereas Hsp86 and Hsp110 were induced only at the higher doses. Induction of Hsp25 and Hsp70 was strain specific; induction by WY was observed in SV129 mice [31] but not in B6C3F1 mice (data not shown). Like ACO-A and ACO-B proteins, induction of Tcp1*η*, Hsp70, Hsp86, and Hsp110 protein expression was observed after exposure to DEHP in wild-type but not PPAR*α*-null mice (Figure 5(b)). These results demonstrate that diverse PPC induce protein chaperone expression dependent on PPAR*α*.

### 3.5. Comparison of the Proteome Maintenance Genes Altered by Chemicals That Activate Other Nuclear Receptors

**Figure 6:**
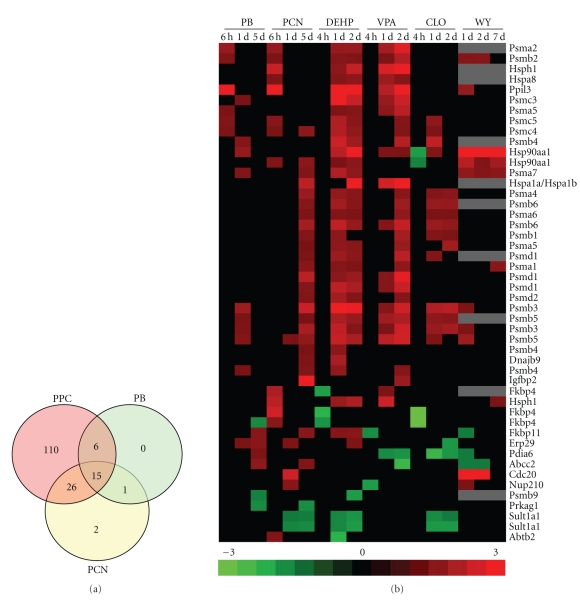
Comparison of the expression of the PM genes after exposure to PPAR*α*, CAR and PXR activators. Lists of differentially regulated genes were generated after exposure to phenobarbital (PB), pregnenolone-16alpha-carbonitrile (PCN) or the PPC. The expression of the PM genes (probe sets) were compared between the treatments. (a) Overlap in the PM gene probesets that were altered in one or more of the three time points after PB or PCN exposure or after 3 or 4 of the 4 PPC treatment groups are shown. (b) The 47 overlapping gene probe sets were clustered as described above.

Many of the PM genes have been shown to be induced under conditions of stress leading to the hypothesis that the response to PPC may be due to activation of a generalized stress response. If that was the case, we would predict that the genes would be altered by other chemicals given at relatively high doses. To determine the specificity of the PPC response, the expression changes of the PM genes were compared between PPC and chemicals which activate other nuclear receptors: phenobarbital (PB) which activates CAR and pregnenolone-16alpha-carbonitrile (PCN) which activates the pregnane X receptor (PXR). In a published study [29], PB, or PCN were given to rats at 100 mg/kg/day for 6 hrs, 1 day or 5 days. Microarray analysis was performed using the same microarray platform and analysis procedures as described above. We compared the PM genes and found that out of the genes that were altered by PPC, PB or PCN, most of the genes were uniquely altered by the PPC (Figure 6(a)). In contrast, only 3 genes were altered by PB and/or PCN but not any of the PPC. There were 47 overlapping genes which exhibited for the most part similar expression by the PPC and PB or PCN (Figure 6(b)). In particular, there was a group of genes that were consistently upregulated by PB, PCN and two or more PPC including *Ppil3, Psma2, Psma7, Psmb2, Psmb3, Psmb4, Psmb5, Psmc4, *and *Psmc5. *Due to their promiscuous induction by most of the chemicals, we hypothesize that these genes were altered due to a generalized stress response and not due to activation of a particular nuclear receptor. However, most of the PM genes that were altered by PPC formed a unique group that was only altered by one or more PPC but not by activators of other nuclear receptors.

## 4. Discussion

The nuclear receptor PPAR*α* is considered a key factor in lipid homeostasis. There is increasing evidence that PPAR*α* plays additional functional roles in the liver by regulating responses to various chemical and physical stressors [14]. An agonist of PPAR*α* (WY) regulates responses in the mouse liver to chemical stress in part by altering expression of genes involved in proteome maintenance (PM) such as the *Hsp* genes involved in protein folding and *Psm* genes involved in proteolysis. In this study, we show that other PPAR*α* activators including diverse hypolipidemic and xenobiotic compounds also regulate a common set of PM genes in the rat and mouse liver. These transcriptional changes were, for the most part dependent on PPAR*α* because most of the altered genes were altered by PPC but not by chemicals that activate other nuclear receptors, CAR and PXR. In mice the changes in the PM genes were observed in wild-type but not PPAR*α*-null mice. The responsive PM genes did not exhibit the same transcriptional behavior as genes known to be direct targets of PPAR*α* (e.g., *Acox1* or *Cyp4a* family members) in which PPAR*α* binds directly to peroxisome proliferator response elements (PPRE) in their promoters. While the direct targets were upregulated early after exposure and remained elevated throughout the duration of the experiment, the PM genes exhibited a lag before expression changed and in many cases, the changes were transient. A number of PM genes were identified that were not universally regulated by all of the PPC. Discordance in the expression pattern between the PPC could be explained in part due to the selection of dose and time which can both influence the gene expression results of these studies. However, our studies indicate that the PM genes are a unique subset of PPAR*α* target genes that appear to be regulated by a mechanism different than fatty acid oxidation genes. 

How are the PM genes regulated by PPC through PPAR*α*? Given that *Hsp* gene expression is controlled in part by heat shock factor 1 (HSF1), one possibility is that the increases in *Hsp* gene expression are secondary to increases in the expression and activity of HSF1. However, we did not observe changes in HSF1 expression in our transcript profiling studies and earlier studies showed that HSF1 and HSF2 expression and binding to HSE were not altered by WY exposure in the rat liver [32]. To help determine whether regulation of *Hsp* gene expression is direct or indirect, we examined their promoters and found that only a few genes possess a putative PPRE(s) (data not shown). The fact that most of the *Hsp* and *Psm* genes are not immediately increased by PPC exposure indicates that additional molecular events are required before induction can occur. 

Many *Hsp* genes may be regulated indirectly through generalized stress responses especially those that are induced after exposure to the relatively high doses of the chemicals used in the animal studies. One of the stress responses that may be driving the expression of the genes is increases in oxidative stress. There is abundant evidence for the increased expression of chaperone gene expression by oxidative stress [10, 33]. PPC exposure leads to increases in oxidative stress and lipid peroxidation mediated through increased activities of enzymes that generate reactive oxygen species (reviewed in [2]). Furthermore, as oxidative stress after PPC exposure is a relatively high dose phenomenon [2], the induction of Hsp86 and Hsp110 only at high doses is consistent with an indirect mechanism of induction, possibly through increases in oxidative stress. Likewise, induction of *Psm* gene expression may be an adaptation to decreases in the levels of functional proteasomes through damage by oxidative stress. Treatment of cells *in vitro* with proteasome inhibitors increased the expression of a broad range of subunits of the proteasome in diverse species [13, 34] even when less than 50% of the total proteasomal activity was inhibited [13]. Proteasome inhibition resulted in increased expression of 19S and 20S components but decreased expression of *Psmb8* [13], a pattern similar to that observed with PPC. Since direct oxidative modification of the catalytic core subunits of the proteasome inhibits their activities [35], PPC may be increasing the level of oxidized proteins that inhibit the proteasome, triggering compensatory increases in *Psm* genes. Lastly, the absolute increases in expression of some *Hsp* and *Psm* genes was higher in WY-treated mice nullizygous for Nrf2, a transcription factor activated by oxidative stress that regulates genes that dampen oxidative stress. Thus, in the absence of Nrf2, increased levels of oxidative stress may have contributed to the greater increases in the PM genes by WY [16]. Taken together, these results indicate that PPAR*α* may regulate the PM genes secondary to increases in oxidative stress. An alternative hypothesis is that PM genes are induced in response to the demand for folded proteins under conditions of increased protein synthesis during active reprogramming of gene expression coincident with increases in peroxisomes and smooth endoplasmic reticulum (SER) and increases in cell number. This hypothesis is consistent with the fact that a subset of the PM genes were induced by CAR, PXR as well as PPAR*α* activators, all of which induce SER proliferation and hepatocyte proliferation. This would also help to explain the somewhat transient nature of the gene expression changes as after acute exposure the liver reaches a new equilibrium in which hepatocyte proliferation returns to normal levels. Further experiments are needed to determine the molecular basis for the induction of the PM genes.

What might the increased levels of PM proteins be doing in the liver after WY exposure? Increased levels of PM gene products might allow tight control of the inducibility of PPAR*α*. Many nuclear receptors interact with chaperone proteins including the ones induced by PPC in our studies [36]. PPAR*α* interacts with Hsp72 [37] and is inhibited by Hsp90 [38]. PPAR*α* activation is also downregulated by proteasomal proteolysis [39]. Thus, induction of some PM genes may dampen the PPAR*α* transcriptional response. *Hsp* induction may also help support the increases in protein synthesis required for liver enlargement including peroxisome proliferation after PPC exposure. Increased expression of TCP1 subunits may be important for proper protein insertion into the peroxisomal membrane [40]. Increased expression of *Hsp* family members has been mechanistically linked to protection from apoptosis [41, 42] and PPC, at least under acute exposure conditions, decrease basal levels of apoptosis [2]. 

A fundamental question arises as to why PPAR*α* would have a dual role of regulating both PM genes involved in stress responses and lipid metabolism genes. The ability of PPAR*α* to act as a regulator of responses to different types of stressors may have coevolved and become inexorably linked with the most important stressor mammals face in the wild, that is, an inadequate or inconsistent food supply. Periods of starvation or caloric restriction requires a reprogramming of gene expression to utilize stored fat and to allow adaptation to new, potentially toxic food sources. PPAR*α* is a master regulator of the starvation response. Gene expression changes by fasting [43, 44] or caloric restriction [14, 17] partly depend on PPAR*α* including genes that mobilize, transport and catabolize fats. The ability of PPAR*α* to also regulate genes (e.g., *Hsp* family members) involved in suppression of cytotoxicity induced by unfolded proteins would make teleological sense and may allow increased resistance to potentially toxic foods animals are forced to eat when the customary foods are no longer available. 

In summary, we used transcript profiling to show that PPAR*α* activated by diverse PPC regulates the expression of two classes of genes that may be responsible for protection from chemical-induced oxidative stress: the chaperone genes involved in protein folding and genes involved in proteasomal degradation of damaged proteins. Induction of these potentially protective pathways may provide efficient means for cells to survive conditions of oxidative stress that contribute to chronic diseases. Induction of these pathways through pharmacological means provides opportunities for protection in a number of settings in which there is induction of oxidative stress, oxidative damage to proteins, and increased occurrence of disease.

##  Author Contributions

H. Ren analyzed the microarray data and helped draft the paper. B. Vallanat performed the microarray data analysis. H. M. B. -Borg analyzed the western data. R. Currie generated microarray data. J. C. Corton conceived of the study, participated in study design and animal studies, generated western data, analyzed microarray data and helped to draft the paper. All authors read and approved the final paper.

## Supplementary Material

Supplemental File 1. Proteome maintenance genes from rats examined in this study. Excel worksheet with genes altered by one or more of the indicated treatments. Information about the genes is included including fold-changes.
Supplemental File 2. Proteome maintenance genes from mice examined in this study. Excel worksheet with genes altered by one or more of the indicated treatments. Information about the genes is included including fold-changes.

## Figures and Tables

**Table 1:** Characteristics of the studies used in the rat and mouse liver microarray analysis.

Species	Reference	GEO Accession number	Strain	Dose frequency	Chemical and (Dose)^1^	Time of treatment	Vehicle	Array type	Number of biological replicates	Total number of .cel files
Rat	Martin et al., 2007 [22]	GSE14712	SD (Wistar)	Once daily	PFOA (20) and PFOS (10)	3 days	15% Alkamuls	RAE230 2.0	3	9
Rat	Ellinger-Ziegelbauer et al., 2005 [28]	GSE14712	SD (Wistar)	Once daily	WY (60)	1 day, 3 days, 7 days	Carboxymethylcellulose	RAE230A	3	12
Rat	Jolly et al., 2005 [21]	GSE2303	SD	Once	DEHP (20,000) Valproic acid (2000)	4, 24, 48 hrs	Distilled water	Rat U34A	3–5	16 (DEHP) 15 (VPA)
Rat	Jolly et al., 2005 [21]	GSE2303	SD	Once	Clofibrate (1000)	4, 24, 48 hrs	0.9% saline	Rat U34A	3–5	16
Rat	Nelson et al., 2006 [29]	GSE14712	SD	Once daily	PB (100) and PCN (100)	6 hrs, 1 day, 5 days	1% Tween-80 in 0.5% aqueous methylcellulose	RAE230 2.0	4	36
Mouse	Sanderson et al., 2008 [23]	GSE8292, GSE8295	SV129 and PPAR*α*-null	Once daily	WY^2^	6 hrs, 5 days		Mouse 430_2	3–5 (6 hrs) 4 (5 days)	17 (6 hrs) 16 (5 days)
Mouse	Currie et al., 2005 [30]	Not submitted	C57BL/6J	Once daily	DEHP (1150)	2, 8, 24 hrs, 3 days	Corn oil	Mouse 430_2	3	24
Mouse	Rosen et al., 2008 [24]	GSE9786	SV129 and PPAR*α*-null	Once daily	PFOA (3)	7 days	Distilled water	Mouse 430_2	4	16
Mouse	Sanderson et al., 2008 [23]	GSE8295 GSE8396	SV129 and PPAR*α*-null	Once	Fenofibrate^2^	6 hrs		430_2	4	16

All exposure experiments were conducted on male rats or mice by gavage.

^1^All doses are in mg/kg/day.

Abbreviations: DEHP: di-2-ethylhexyl phthalate; WY: WY-14,643; PFOA: perfluorooctanoic acid; PFOS: perfluorooctane sulfonate; SD: Sprague-Dawley; PB: phenobarbital; PCN: pregnenolone-16alpha-carbonitrile.

^2^400 *μ*L of a 10 mg/mL solution/day.

## Abbreviations

CLO: clofibrate;
DEHP: di-(2-ethylhexyl)phthalate;
FENO: fenofibrate;
HSF: heat shock factor;
HSP: heat shock proteins;
PCR: polymerase chain reaction;
PFOA: perfluorooctanoic acid;
PFOS: perfluorooctane sulfonate;
PM: protein maintenance;
PPAR: peroxisome proliferator-activated receptor;
PPC: peroxisome proliferator chemical;
PPRE: peroxisome proliferator response element;
Psm: proteasome;
VPA: valproic acid;
WY: WY-14,643.
